# A glutaminyl cyclase-catalyzed α-synuclein modification identified in human synucleinopathies

**DOI:** 10.1007/s00401-021-02349-5

**Published:** 2021-07-26

**Authors:** Maike Hartlage-Rübsamen, Alexandra Bluhm, Sandra Moceri, Lisa Machner, Janett Köppen, Mathias Schenk, Isabel Hilbrich, Max Holzer, Martin Weidenfeller, Franziska Richter, Roland Coras, Geidy E. Serrano, Thomas G. Beach, Stephan Schilling, Stephan von Hörsten, Wei Xiang, Anja Schulze, Steffen Roßner

**Affiliations:** 1 Paul Flechsig Institute for Brain Research, University of Leipzig, Liebigstraße 19, 04103 Leipzig, Germany; 2 Department for Experimental Therapy, Universitätsklinikum Erlangen, and Preclinical Experimental Animal Center, Friedrich-Alexander-Universität Erlangen-Nürnberg, 91054 Erlangen, Germany; 3 Department of Molecular Drug Design and Target Validation, Fraunhofer Institute for Cell Therapy and Immunology, 06120 Halle (Saale), Germany; 4 Department Molecular Neurology, Universitätsklinikum Erlangen, Friedrich-Alexander-Universität Erlangen-Nürnberg, 91054 Erlangen, Germany; 5 Department of Pharmacology, Toxicology, and Pharmacy, University of Veterinary Medicine Hannover, 30559 Hannover, Germany; 6 Institute for Neuropathology, Universitätsklinikum Erlangen, Friedrich-Alexander-Universität Erlangen-Nürnberg, 91054 Erlangen, Germany; 7 Civin Laboratory for Neuropathology Brain and Body Donation Program Banner Sun Health Research Institute, 10515 W Santa Fe Drive, Sun City, AZ 85351 USA

**Keywords:** α-Synuclein, Post-translational modification, Parkinson’s disease, Dementia with Lewy bodies, Glutaminyl cyclase, Substantia nigra, Animal models

## Abstract

Parkinson’s disease (PD) is a progressive neurodegenerative disorder that is neuropathologically characterized by degeneration of dopaminergic neurons of the substantia nigra (SN) and formation of Lewy bodies and Lewy neurites composed of aggregated α-synuclein. Proteolysis of α-synuclein by matrix metalloproteinases was shown to facilitate its aggregation and to affect cell viability. One of the proteolysed fragments, Gln79-α-synuclein, possesses a glutamine residue at its N-terminus. We argue that glutaminyl cyclase (QC) may catalyze the pyroglutamate (pGlu)79-α-synuclein formation and, thereby, contribute to enhanced aggregation and compromised degradation of α-synuclein in human synucleinopathies. Here, the kinetic characteristics of Gln79-α-synuclein conversion into the pGlu-form by QC are shown using enzymatic assays and mass spectrometry. Thioflavin T assays and electron microscopy demonstrated a decreased potential of pGlu79-α-synuclein to form fibrils. However, size exclusion chromatography and cell viability assays revealed an increased propensity of pGlu79-α-synuclein to form oligomeric aggregates with high neurotoxicity. In brains of wild-type mice, QC and α-synuclein were co-expressed by dopaminergic SN neurons. Using a specific antibody against the pGlu-modified neo-epitope of α-synuclein, pGlu79-α-synuclein aggregates were detected in association with QC in brains of two transgenic mouse lines with human α-synuclein overexpression. In human brain samples of PD and dementia with Lewy body subjects, pGlu79-α-synuclein was shown to be present in SN neurons, in a number of Lewy bodies and in dystrophic neurites. Importantly, there was a spatial co-occurrence of pGlu79-α-synuclein with the enzyme QC in the human SN complex and a defined association of QC with neuropathological structures. We conclude that QC catalyzes the formation of oligomer-prone pGlu79-α-synuclein in human synucleinopathies, which may—in analogy to pGlu-Aβ peptides in Alzheimer’s disease—act as a seed for pathogenic protein aggregation.

**Supplementary Information:**

The online version contains supplementary material available at 10.1007/s00401-021-02349-5.

## Introduction

Parkinson’s disease (PD) is the second most frequent progressive neurodegenerative disorder after Alzheimer’s disease (AD) [7, 12]. The brains of PD patients are neuropathologically characterized by the degeneration of dopaminergic neurons of the substantia nigra (SN) pars compacta, which results in dopamine depletion of the striatum [52, 68]. This dopaminergic hypoactivity affects functions of the complex basal ganglia network, leading to clinical symptoms, such as hypokinesis and tremor [15, 34]. Another typical feature of PD is the appearance of Lewy bodies and Lewy neurites that are mainly composed of aggregated α-synuclein [28, 82]. Under physiological conditions, α-synuclein is believed to be a natively unfolded protein of 140 amino acids, but it may also exist as α-helically folded multimers [8, 25]. It is predominantly localized to presynaptic nerve terminals and has been shown to act as a molecular chaperone in the formation of SNARE complexes being involved in the regulation of dopamine release [35]. In the course of PD, however, α-synuclein conformation is altered to form Lewy inclusions and various aggregation conformers, ranging from small oligomers to amyloid fibrils, with distinct structural and biochemical features [5, 59, 100]. Recent studies provided evidence that aggregated α-synuclein may propagate its structural alterations and loss or gain of function via prion-like spreading [47, 54, 60, 106]. Similarly, α-synuclein aggregates are also present in brains of patients suffering from multiple systems atrophy and from dementia with Lewy bodies (DLB), where the pathology also affects cortical association areas [40, 75, 101].

**Fig. 1:**
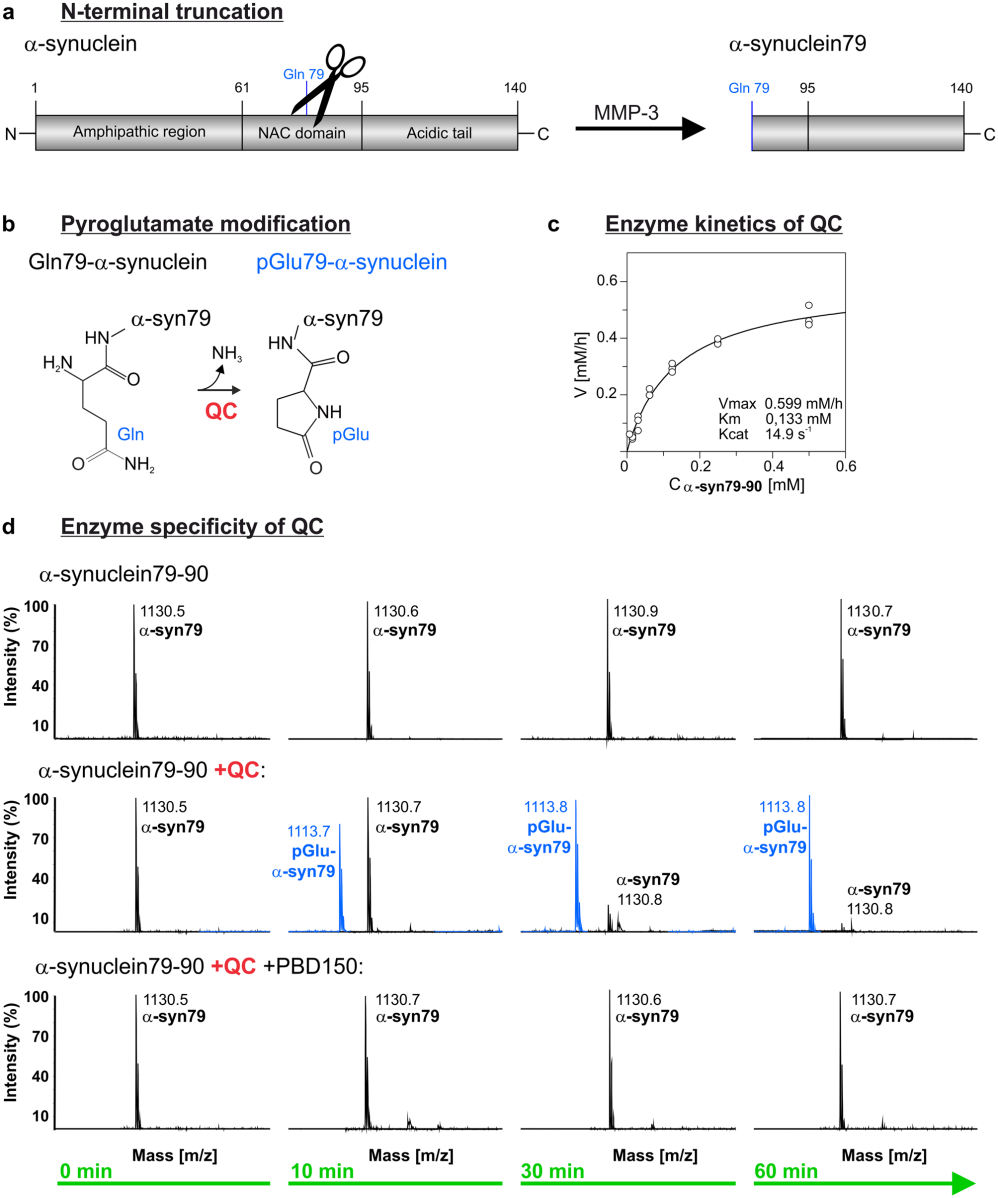
QC-catalyzed pGlu79-α-synuclein formation. **a** Schematic representation of N-terminal α-synuclein truncation by MMP-3 resulting in the formation of N-terminal glutamine (Gln) residue at position 79 of α-synuclein. **b** Schematic illustration of pGlu formation from N-terminal Gln under liberation of ammonia catalyzed by QC. **c** Kinetic characteristics of QC-catalyzed pGlu79-α-synuclein formation revealed by a continuous coupled spectrophotometric test. Values were obtained from 3 to 4 independent determinations and are displayed as mean ± SD. **d** Mass spectrometric analysis of pGlu formation at the N-terminus of the synthetic α-synuclein79–90 fragment. The maternal α-synuclein79–90 fragment was detected at the predicted mass of 1130.6 Da at all time points without any spontaneous degradation or modification. When the enzyme QC was added, a new peak (blue) was detected at a molecular weight of 17 g/mol below the maternal α-synuclein79–90 fragment, consistent with the liberation of ammonia during enzyme-catalyzed pGlu-formation. The conversion of α-synuclein to pGlu-α-synuclein79-90 was completely prevented by addition of the QC inhibitor PBD150

Structurally, α-synuclein is composed of 3 domains: an amphipathic N-terminal region (aa 1–60), a central hydrophobic domain involved in protein aggregation (non-Abeta component (NAC) region; aa 61–95) and a highly acidic, proline-rich C-terminus (aa 96–140) [33]. The full-length α-synuclein can be post-translationally modified by phosphorylation, ubiquitination, nitration, glycation, SUMOylation and truncation [4, 37, 105, 109]. C-terminal truncations of α-synuclein by defined protease activities, such as m-calpain and 20S proteasome, have been linked to increased aggregation, fibril formation and neurotoxicity [55, 63, 74, 99]. In addition, N-terminally truncated α-synuclein fragments are generated by matrix metalloproteinases (MMPs) -1, -3 and -9 [61, 102]. Most importantly, limited proteolysis of α-synuclein by MMP-1 and MMP-3, but not by MMP-9, was shown to generate fragments that increase de novo aggregation of α-synuclein in vitro [61]. Since α-synuclein is cleaved by MMP-3 preferentially within the NAC domain, the resultant fragments do not form fibrils but rather oligomers that compromise cell viability [102]. One of the MMP-3-generated α-synuclein fragments, Gln79-α-synuclein, possesses a glutamine residue at its N-terminus (Fig. 1a).

Peptides with an N-terminal glutamate or glutamine residue may serve as substrates for glutaminyl cyclase (QC), giving rise to pyroglutamate (pGlu)-modified peptides [92, 97]. This pGlu modification confers stability against proteolytical degradation and increases the biological activity of neuropeptides and peptide hormones, such as orexin A, gastrin, gonadotropin- and thyrotropin-releasing hormones and neurotensin in hypothalamus and pituitary [14, 18, 29, 81].

Under pathological conditions in AD, however, QC catalyzes the pGlu modification of N-truncated Aβ peptides that are highly pathogenic and act as seeds for Aβ oligomer and plaque formation [3, 23, 76, 93]. QC expression is developmentally regulated [43] and highly abundant in brain structures affected by amyloid pathology in AD, such as nucleus basalis Meynert, locus coeruleus and Edinger–Westphal nucleus [73], hippocampus [42] and neocortex [72]. Pharmacological inhibition of QC activity [93] and genetic ablation of QC in experimental animal models [3, 50] reduced pGlu-Aβ generation and total Aβ load and ameliorated learning and memory deficits. It is tempting to speculate that QC—if expressed by dopaminergic SN neurons—may catalyze the pGlu79-α-synuclein formation and, thereby, contribute to enhanced aggregation and compromised degradation of α-synuclein in human synucleinopathies (Fig. 1b).

Therefore, we here analyzed the enzymatic formation of pGlu79-α-synuclein by QC in vitro, its aggregation characteristics and neurotoxic profile, its co-localization with QC and increased formation in the SN of human PD and DLB brains as well as in animal models for synucleinopathies. Together, we demonstrate the existence of a novel pathogenic post-translational α-synuclein modification. Since this modification is QC-catalyzed and QC inhibitors are already in clinical trials for AD treatment, there might be novel therapeutic options for interfering with α-synuclein aggregation in PD as well.

## Materials and methods

### QC-catalyzed formation of pGlu79-α-synuclein

#### Peptide synthesis

The synthetic α-synuclein79–90 peptide was synthesized according to standard Fmoc solid phase protocols on a Tetras peptide synthesizer (Advanced ChemTech, Louisville, USA) at 60 µmol scale as C-terminal amide on Rink amide resin (Iris Biotech; Marktredwitz, Germany) using standard Fmoc/tBu-protected amino acids (Iris Biotech). Coupling was done using *O*-(benzotriazol-1-yl)-*N*,*N*,*N*',*N*′-tetramethyluronium tetrafluoroborate (TBTU) and *N*-methylmorpholine (NMM). Fmoc-deprotection was carried out using 20% piperidine in DMF. Final cleavage and deprotection of the peptides was performed using TFA:EDT:H_2_O:TIS (50:2:2:1 v/v). After precipitation with cold diethylether, the peptides were purified by preparative reversed phase (RP)–HPLC (Phenomenex Luna C18(2) column) and eluted with an increasing water:acetonitrile gradient starting with 5% containing 0.04% TFA. The identity and purity were assessed by analytical RP–HPLC and ESI MS.

#### Enzymatic activity assay

The kinetics of QC-catalyzed pGlu-α-synuclein79–90 formation was measured by a continuous coupled spectrophotometric test in 50 mM Tris/HCl buffer, pH 8.0 using glutamate dehydrogenase as auxiliary enzyme as described in detail by Schilling et al. [90, 91]. Kinetic parameters were calculated by non-linear regression as indicated before [95].

#### Mass spectrometry

The pGlu formation at the N-terminus of the synthetic α-synuclein79–90 peptide was monitored by mass spectrometry. 22.6 µg of this peptide were incubated in a total volume of 200 µl 50 mM Tris/HCl buffer, pH 8.0, (100 µM) for 10, 30 and 60 min in the absence or presence of the enzyme QC (0.7 µg/ml; 20 nM) with and without the QC inhibitor PBD150 (100 µM). The analytes were ionized by a nitrogen laser pulse (337 nm) and accelerated under 20 kV with a time-delayed extraction before entering the time-of-flight mass spectrometer (Voyager De Pro, Sciex). The maternal synthetic α-synuclein79–90 peptide was detected at the mass of 1130.6 Da, whereas after pGlu79 modification and liberation of ammonia the molecular weight was reduced to 1113.8 Da.

### Aggregation of recombinant α-synuclein and pGlu79-α-synuclein

#### Expression and purification of α-synuclein proteins

The human full-length α-synuclein and Gln79-α-synuclein proteins were recombinantly expressed following procedures described recently [57]. Purification included Ni^2+^-chelating chromatography on a Streamline Chelating resin (Streamline Chelating, GE Healthcare Life Sciences, Uppsala, Sweden). Fractions containing the expression construct were subjected to a second purification step via a glutathione sepharose resin (Glutathione Sepharose 4FF, GE Healthcare Life Sciences). The removal of glutathione was achieved by overnight dialysis against buffer containing 100 mM NaCl, 30 mM Tris/HCl pH 7.6, 0.1 mM DTT and a membrane with 6–8 kDa cutoff. Separation of the GST- and His-tag from the α-synuclein sequence by a TEV protease cleavage left an native N-terminus [53] followed by cyclization of Gln79-α-synuclein to pGlu79-α-synuclein with QC overnight at room temperature. The fractions obtained were analyzed and subjected to reversed phase chromatography (Source 15 RPC, GE Healthcare Life Sciences), followed by lyophilization and anion exchange chromatography (MonoQ 5/50GL, GE Healthcare Life Sciences). The final buffer used for the experiments was 20 mM Tris/HCl, pH 7.0, containing 100 mM NaCl. The purity of the samples was assessed by SDS PAGE and mass spectrometry. Protein concentrations were determined using UV absorption at 280 nm.

#### Thioflavin T assay

The thioflavin T (ThT) assay was carried out as described previously [94] on a FluoStar Optima (BMG Labtech, Ortenberg, Germany) plate reader using a 96-well plate (*λ*_ex_ = 440 nm and *λ*_em_ = 490 nm). For monitoring the fibrillation process of the recombinant full-length or pGlu79-α-synuclein, 20 µM ThT (Sigma-Aldrich) were added to the aggregation buffer (20 mM Tris/HCl, 100 mM NaCl, pH 7.0). Signals were recorded at 37 °C under continuous shaking (300 rpm) with a time interval of 15 min for 110 h. Analyses of the obtained aggregation curves were conducted according to [46]. For each peptide, measurements were performed in six cavities of one plate. Obtained data were analyzed with one-way ANOVA and post-hoc Tukey test.

#### Transmission electron microscopy

Potential fibril formation from full-length α-synuclein and pGlu79-α-synuclein was initiated in aggregation buffer (20 mM Tris/HCl, 100 mM NaCl, pH 7.0) at 37 °C under continuous shaking (300 rpm) for 110 h. Samples (5 µl) were placed on a formvar carbon-coated copper grid (Plano, Wetzlar, Germany) for 10 min and washed three times with distilled water. Staining was obtained with 2% (v/v) phosphotungstic acid (Sigma-Aldrich) for 5 min. Grids were imaged with a TEM/STEM FEI-Tecnai G2 F20 (FEI Company, Hillsboro, USA) in STEM-mode at 200 kV. The electron micrographs were detected using a high-angle annular dark-field detector, and finally processed by contrast-inversion.

#### Size exclusion chromatography and dot blot analysis

To analyze the formation of oligomers by size exclusion chromatography (SEC), 50–120 µg of the recombinant full-length or pGlu79-α-synuclein, either untreated (monomers) or agitated for aggregation as described in ThT assay, was centrifuged at 10,000×*g* for 60 min to remove large aggregated particles. Centrifuged α-synuclein samples of each variant were next diluted with SEC running buffer (50 mM Tris/HCl pH 7.2 buffer with 200 mM NaCl) to a total volume of 300 µl, filtered by a Whatman PVDF filter device (pore size 0.2 µm) and subsequently loaded onto a Yarra SEC 3000 column (Phenomenex, Aschaffenburg, Germany). SEC was performed using an isocratic elution with the SEC running buffer at a flow rate of 0.3 ml/min on an ÄKTA pure 25 M system (Cytiva, Freiburg, Germany). A total of 30 fractions of each analysis were collected with 0.5 ml per fraction. The eluted peaks were monitored at 215 and 280 nm. The elution time and quantity of the monomers and oligomers were determined using Unicorn software (Cytiva). For immunodetection of the fractions containing α-synuclein, the collected fractions were applied onto a nitrocellulose membrane using a Minifold Dot-Blot System (Schleicher & Schuell) and probed with Syn1 (BD Transduction Laboratories™), a monoclonal mouse antibody against pan α-synuclein (1:1000), or the polyclonal rabbit anti-pGlu79-α-synuclein antibody (described below, 1:700). For immunodetection, horseradish peroxidase conjugated anti-mouse or anti-rabbit antibody (Dianova) and chemiluminescent substrates (SuperSignal West Chemiluminescent Substrate kits, Thermo Fisher Scientific) were used.

#### Cell culture and toxicity assay

The toxic effect of full-length and pGlu79-α-synuclein on SH-SY5Y neuroblastoma cells was assessed using a WST-1 assay (ThermoFisher, Darmstadt, Germany). SH-SY5Y cells were grown in DMEM medium supplemented with 10% FBS at 37 °C, 10% CO_2_. To induce differentiation towards a neuronal phenotype, 1.83 × 10^4^ cells/well were seeded in a transparent 96-well plate and the medium was changed towards DMEM supplemented with 5% FBS and 10 µM all-trans retinoic acid (ThermoFisher, Darmstadt, Germany) for 3 days. The medium was further exchanged to Neurobasal-A medium without phenol red, supplemented with 1% (v/v) Glutamax, 1% (v/v) N-2 supplement (ThermoFisher, Darmstadt, Germany) and human BDNF (ThermoFisher, Darmstadt, Germany) at a concentration of 50 ng/ml (v/v) for additional 4 days. On day 7 of differentiation, the assay was carried out according to the manufacturer’s protocol. In brief, cells were exposed to the different peptide species and cultured at 37 °C in a humidified atmosphere containing 10% CO_2_ for 72 h. Afterwards, 10% WST-1 was added to the cell medium and incubated for 30 min. The absorbance was determined at 440 nm using a plate reader (Tecan Sunrise, Switzerland). The values were normalized to the PBS control and directly correlated to the number of viable cells.

### Mouse brain tissue

The expression of endogenous α-synuclein was analyzed in brains of C57Bl/6 wild-type mice (*N* = 4) obtained from the Animal Care Facility of the Medical Faculty, Leipzig University. α-synuclein knock-out (KO) mice (*N* = 2; Charles River; JAX strain 003692) were used to demonstrate the specificity of α-synuclein antibodies employed in this study. Two transgenic mouse lines were investigated for the formation of α-synuclein aggregates: (1) mice overexpressing human wild-type α-synuclein under the Thy-1 promoter (termed ASO; *N* = 4) [20, 83] and (2) mice overexpressing human wild-type α-synuclein from a bacterial artificial chromosome (termed BAC-SNCA; *N* = 4) [71, 108]. All mouse lines were on C57Bl/6 background. Animals were housed at 12 h day/12 h night cycles with food and water ad libitum in cages that contained nest building material. All experimental protocols were approved by Landesdirektion Sachsen, license number T28/16 and the local ethical board of the District Government of Lower Franconia, Bavaria, Germany (approval # 55.2-DMS 2532-2-218). All methods were carried out in accordance with the relevant guidelines and regulations.

#### Tissue preparation

Mice were sacrificed by CO_2_ inhalation and perfused transcardially with 0.9% saline followed by perfusion with 4% paraformaldehyde in phosphate buffer (0.1 M, pH 7.4). The brains were removed from the skull and post-fixed by immersion in the same fixative overnight at 4 °C. After cryoprotection in 30% sucrose in 0.1 M phosphate buffer for 3 days, 30 µm thick coronal sections were cut on a sliding microtome and collected in phosphate buffer supplemented with 0.025% sodium azide for storage.

### Human brain tissue

#### Case recruitment and characterization of human brain tissue

Case recruitment and autopsy were performed in accordance with guidelines effective at the Arizona Study of Aging and Neurodegenerative Disorders and Brain and Body Donation Program [10]. The required consent was obtained for all cases. Cases were staged for synuclein pathology using the Unified Staging System [2, 9]. The definite diagnosis of PD was based on clinical findings of 2 of 3 cardinal signs (rigidity, bradykinesia and rest tremor) as well as depigmentation with Lewy bodies in the SN. DLB was defined as dementia occurring either at presentation or within 1 year of the onset of parkinsonism, with a brain distribution of α-synuclein pathology meeting DLB Consortium criteria for “intermediate” or “high” likelihood [70]. The Unified Staging System and McKeith criteria received good inter-rater reliability scores in a multi-centre comprehensive analysis defining consensus criteria for the evaluation of Lewy body pathology in post mortem brains [6]. Three out of 10 DLB cases were additionally diagnosed with AD, due to intermediate or high AD neuropathological changes according to [21].

#### Tissue preparation

Transverse midbrain sections (40 µm thick) comprising SN at the level of the red nucleus, exit of the oculomotor nerve and superior colliculus from 10 controls, 10 idiopathic PD cases and 10 DLB cases (Table 1) were used for QC and pGlu79-α-synuclein immunohistochemistry. Anatomical regions were identified on Nissl and anti-HuC/D-stained sections using a human brain atlas [66].

**Table 1:** Cocktails of primary antibodies used for triple labelling immunohistochemistry

Primary antibody	Dilution	Host	Company	Secondary antibody
QC	1:100	Goat	IZI	Donkey anti-goat Cy2
α-Synuclein (Syn1)	1:3000	Mouse	BD transduction	Donkey anti-mouse Cy3
TH	1:200	Guinea pig	Synaptic systems	Donkey anti-guinea pig Cy5
QC	1:100	Goat	IZI	Donkey anti-goat Cy2
pGlu79-α-synuclein	1:100	Rabbit	IZI	Donkey anti-rabbit Cy3
α-Synuclein (Syn1)	1:3000	Mouse	BD transduction	Donkey anti-mouse Cy5

Secondary antibodies were all from Dianova and used at a dilution of 1:200

*TH* tyrosine hydroxylase, *QC* glutaminyl cyclase

### Antibody generation

Polyclonal anti-pGlu79-α-synuclein-specific antibodies were produced from rabbits immunized with synthetic pGlu-α-synuclein79–90 peptide conjugated to a carrier according to the manufacturer’s standard protocol (Davids Biotechnology, Germany). Rabbits were immunized five times (days 1, 14, 28, 42 and 56) with the optimal amount of antigen followed by a final bleed at day 63. After day 35, a test serum was taken and the ELISA titer was determined. The antiserum was affinity purified and characterized for specificity (Suppl. Figure 1, online resource).

#### Antibody specificity

The specificity of the rabbit antiserum against pGlu79-α-synuclein was verified by dot blot analysis against recombinant human full-length α-synuclein, β-synuclein, γ-synuclein and the target pGlu79-α-synuclein fragment, spotted at descending amounts onto nitrocellulose membranes (Suppl. Figure 1, online resource). After chemiluminescent detection, membranes were stripped and re-probed with the Syn1 antibody (BD Transduction; 1:2000). In addition, immunohistochemistry was performed on wild-type, α-synuclein overexpressing and α-synuclein KO mouse brain sections, demonstrating the specificity of the pGlu79-α-synuclein antiserum for this application (Suppl. Figure 1, online resource). The specificity of the goat antiserum directed against QC has been recently demonstrated comparing immunohistochemical labelling in wild type and QC KO mouse brain sections [41].

### Immunohistochemistry

#### Single labelling of pGlu79-α-synuclein and QC in mouse brain sections

To detect pGlu79-α-synuclein and QC in wild type, ASO and BAC-SNCA mice, single labelling immunohistochemistry was performed on free-floating coronal brain sections. Brain sections were washed in 0.1 M phosphate buffer (pH 7.4) for 5 min and endogenous peroxidases were inactivated by treating brain slices with 60% methanol containing 1% H_2_O_2_ for 60 min followed by three washing steps with Tris buffered saline (TBS, 0.1 M, pH 7.4) for 5 min each. After masking unspecific binding sites with blocking solution (5% normal donkey serum in TBS containing 0.3% Triton X-100) for 60 min, sections were incubated with the primary rabbit anti-pGlu79-α-synuclein (1:200) or goat anti-QC (1:200) antibodies for 40 h at 4 °C. Brain sections were then washed three times in TBS for 5 min each before being incubated with biotinylated secondary donkey anti-goat or donkey anti-rabbit antibodies (Dianova; 1:1000) in TBS containing 2% bovine serum albumin (BSA) for 60 min. After three washing steps in TBS for 5 min each, slices were incubated with ExtrAvidin peroxidase (Sigma; 1:2000) in TBS/2% BSA followed by washing steps and pre-incubation in Tris buffer (0.05 M, pH 7.6) for 5 min. Finally, visualization of peroxidase binding was performed by incubation with 4 mg 3,3′-diaminobenzidine (DAB) and 2.5 µl 30% H_2_O_2_ per 5 ml Tris buffer. After washing, sections were mounted onto glass slides and cover slipped.

#### Triple immunofluorescent labellings in mouse brain

To reveal the expression of QC by tyrosine hydroxylase (TH)-positive dopaminergic SN neurons and its possible co-localization with full-length α-synuclein and with pGlu79-α-synuclein in mouse SN, the goat anti-QC antibody was applied in cocktails with primary guinea pig antibodies against TH (Synaptic Systems; #213104), mouse anti-α-synuclein (Syn1; BD Transduction Laboratories) or rabbit anti-pGlu79-α-synuclein as specified in Table 1. Brain sections were incubated with cocktails of primary antibodies for 40 h at 4 °C. Sections were then washed three times with TBS followed by incubation with cocktails of Cy2-, Cy3- or Cy5-conjugated donkey anti-mouse, -rabbit, -guinea pig or -goat, respectively, antisera (1:200 each; Dianova) in TBS containing 2% BSA for 60 min at room temperature. After washing, sections were mounted onto glass slides and cover slipped. Switching the fluorescent labels of the secondary antibodies generated similar results as when following the procedure outlined above (not shown).

#### Detection of pGlu79-α-synuclein and QC in human SN

To reveal presence of pGlu79-α-synuclein and QC in the SN of *post mortem* human control, DLB and PD tissue, single labelling immunohistochemistry was performed on free-floating transverse midbrain sections. Sections were washed in phosphate buffered saline (PBS, pH 7.4) for 5 min and endogenous peroxidases were inactivated by treating brain slices with 60% methanol containing 1% H_2_O_2_ for 30 min followed by rinses with PBS containing 0.02% Tween 20 (PBS-T) for 5 min each. Unspecific staining was then blocked in PBS-T containing 2% BSA, 0.3% milk powder and 0.5% normal donkey serum before incubating brain sections in the same solution containing the primary antibodies rabbit anti-pGlu79-α-synuclein (1:200) or goat anti-QC (1:200) in a humid chamber for 40 h at 4 °C. Subsequently, sections were washed in PBS-T (three times for 5 min) and were then incubated with secondary biotinylated donkey anti-rabbit or donkey anti-goat antibodies (Dianova; 1:1,000) in a mixture of blocking solution and PBS-T (1:2) for 60 min at room temperature. Following washing steps, the ABC method was applied which comprised incubation with complexed streptavidin and biotinylated horseradish peroxidase (Sigma; 1:2000) in PBS-T. Binding of peroxidase was visualized by incubation with 2 mg DAB, 20 mg nickel ammonium sulfate and 2.5 µl 30% H_2_O_2_ per 5 ml Tris buffer (0.05 M; pH 8.0) for 3–4 min. DAB-Ni staining resulted in black visualization of pGlu79-α-synuclein and QC which allowed for the co-localization with brown, neuromelanin-positive (NM^+^) neurons in the SN.

For all single and triple immunohistochemical labellings in brain sections described above, control experiments in the absence of primary antibodies were carried out. In each case, this resulted in unstained brain sections (not shown).

### Microscopy

#### Light microscopy

Mouse and human brain tissue sections immunohistochemically stained with DAB or DAB-Ni for pGlu79-α-synuclein and QC expression were examined with an Axio-Scan.Z1 slide scanner connected with a Colibri.7 light source and a Hitachi HV-F202SCL camera (Carl Zeiss, Göttingen, Germany). High resolution images of midbrain sections containing the SN were taken using a 20× objective lens with 0.5 numerical aperture (Zeiss). Images were digitized by means of ZEN 2.6 software and analyzed using the ZEN imaging tool.

#### Confocal laser scanning microscopy

Laser scanning microscopy (LSM 880 Airyscan, Zeiss, Oberkochen, Germany) using an Axioplan2 microscope was performed to reveal co-localization of QC with its substrate pGlu79-α-synuclein, with full-length α-synuclein and with TH, respectively. For Cy2-labelled antigens (green fluorescence), an argon laser with 488 nm excitation was used and emission from Cy2 was recorded at 510 nm applying a low-range band pass (505–550 nm). For Cy3-labelled antigens (red fluorescence), a helium–neon laser with 543 nm excitation was applied and emission from Cy3 at 570 nm was detected applying high-range band pass (560–615 nm) and Cy5-labelled antigens (blue fluorescence) were detected using excitation at 650 nm and emission at 670 nm. Images of areas of interest were taken using a 20× objective lens with 0.75 numerical aperture (Zeiss). Photoshop CS2 (Adobe Systems, CA) was used to process the images obtained by light and confocal laser scanning microscopy. Care was taken to apply the same brightness, sharpness, color saturation and contrast adjustments in the various pictures.

### Quantification of QC staining in SN of human midbrain

Analysis of QC expression in the SN pars compacta (SNc) from control subjects (CO), as well as from DLB and PD patients (*N* = 10, each; Table 2) was performed using the ZEN 2.6 imaging software. Transverse sections (40 µm) of the ventral midbrain approx. at the level of the center of Ncl. ruber were evaluated with respect to intra- and extracellular QC immunoreactivity. For each individual case, the area of the SNc was delineated at lower magnification according to the distribution of the nigral matrix and nigrosomes, respectively, enclosing pigmented NM-containing neurons in the SNc [24]. Hereby, even at high zoom levels, orientation within the SN was assured to restrict examination of QC immunoreactive structures to the pars compacta subregion.

**Table 2:** Human brain tissue (abbreviations see below)

Case	PMI	Sex	Age (years)	Brain weight (g)	MMSE	UPDRS	Lewy stage	SN	Braak NF B Score	Amyloid phase A Score	Neuritic plaque C Score	NIA-AA	Clinicopathological diagnosis		
													AD	PD	DLB
Controls															
1	2.75	M	81	1190	25	6.5	0	None	III (B2)	5 (A3)	C1	Int	No	No	No
2	2.16	M	80	1140	30	3	0	None	I (B1)	5 (A3)	C0	Low	No	No	No
3	2.25	F	82	940	30	4	0	None	II (B2)	0 (A0)	C0	Not	No	No	No
4	1.5	M	91	1440	27	11	0	None	III (B3)	2 (A1)	C0	Int	No	No	No
5	2.16	M	82	1160	28		0	Mild	II (B1)	1 (A1)	C1	Low	No	No	No
6	2.5	M	73	1240	30	4	0	Mild	III (B2)	1 (A1)	C1	Int	No	No	No
7	2.75	M	78	1200	28	8.5	0	None	II (B1)	2 (A1)	C1	Low	No	No	No
8	1.5	M	97	1260	30	13	0	None	III (B3)	2 (A1)	C0	Int	No	No	No
9	2.5	F	95	1298	28	8	0	None	III (B3)	(A0)	C0	Not	No	No	No
10	3.0	M	79	1428	29	3	0	None	III (B3)	0 (A0)	C0	Not	No	No	No
Mean	2.31	8/2	83.8	1230	28.5	6.8									
PD															
1	2.75	F	82	1215	ND	60	III	Severe	IV (B3)	3 (A2)	C2	Int	No	Yes	No
2	2.16	F	73	1170	19	78	IV	Severe	IV (B2)	3 (A2)	C1	Int	No	Yes	No
3	1.83	M	70	1300	8	30.5^@^	IIIc	Severe	III (B2)	2 (A1)	C0	Low	No	Yes	No
4	2.0	M	85	1460	28	12^@^	III	Severe	III (B2)	ND	C2	ND	No	Yes	No
5	3.5	F	78	1120	18	68^@^	IV	Severe	IV (B2)	ND	C1	ND	No	Yes	No
6	2.16	M	85	1320	27	6	IIb	None	III (B2)	3 (A2)	C2	Int	No	Yes	No
7	2.66	M	89	1200	29	0	IIa	Mild	IV (B2)	4 (A3)	C2	Int	No	Yes	No
8	2.0	F	84	1220	21	39.5^@^	IIa	Severe	II (B1)	2 (A1)	C0	Low	No	Yes	No
9	3.5	M	72	1360	25	31.5^@^	III	Severe	II (B1)	ND	C0	ND	No	Yes	No
10	4.16	M	69	1210	24	21	III	Severe	III (B2)	ND	C0	ND	No	Yes	No
Mean	2.67	6/4	78.7	1258	22.1	34.6									
DLB															
1	3.33	M	78	1300	ND	65	IV	Severe	I (B1)	3 (A2)	C2	Low	Yes	No	Yes
2	2.5	F	90	960	24		III	Mild	III (B2)	1 (A1)	C1	Low	No	No	Yes
3	3.75	M	87	1160	22		III	Moderate	II (B1)	0 (A0)	C0	Not	No	No	Yes
4	2.66	F	82	1075	5	72	IV	Moderate	V (B3)	2 (A1)	C3	Int	Yes	No	Yes
5	2.3	M	86	1130	10	22	IV	Moderate	V (B3)	ND	C3	ND	Yes	No	Yes
6	3.16	F	96	1195	ND		III	None	III (B2)	0 (A0)	C0	Not	No	No	Yes
7	3.33	M	89	1250	16		IV	Moderate	III (B2)	2 (A1)	C1	Low	No	No	Yes
8	2.0	M	89	1310	ND		Int*	ND	III (B2)	ND	C1	ND	No	No	Yes
9	2.5	M	89	1225	ND		Int*	ND	III (B2)	ND	C1	ND	No	No	Yes
10	2.0	M	81	1280	26		Neo*	ND	V (B3)	ND	C2	ND	No	No	Yes
Mean	2.75	7/3	86.7	1188	17.2	53.0									

*PMI* postmortem interval in hours, *M* male, *F* female, *MMSE* last Mini Mental State Examination score before death, *UPDRS* Unified Parkinson’s Disease Rating Scale Part 3 (motor), *@* on medication, all others are off medication; Lewy Stage is by the Unified System, or McKeith where indicated by *; *Int* intermediate, *Neo* McKeith neocortical, *Unified Stage I* Olfactory bulb only, *IIa* Brainstem predominant, *IIb* Limbic predominant, *III* Brainstem and limbic, *IV* Neocortical, *SN* substantia nigra pigmented neuron loss, *Braak NF* Braak neurofibrillary stage, *NIA-AA* National Institute on Aging–Alzheimer’s Association AD Neuropatholological Change Level, *ND* not done, *Not* NIA Not AD

#### Evaluation of intracellular QC staining

Zooming from overview to high-resolution magnification in a given specimen, neuronal QC immunoreactivity (DAB-Ni; black) was assessed in each individual neuron throughout the medioventral to dorsolateral extent of the SNc by an investigator blinded to the origin of the case. The presence of intracellular QC, either bound to NM or independently distributed in the cytoplasm, was evaluated with respect to the presence or absence of NM (brown) and vice versa. Then, each neuron was assigned to one of the following three categories: (1) QC and NM positive (QC^+^/NM^+^), (2) QC positive and NM negative (QC^+^/NM^−^), or (3) QC negative and NM positive (QC^−^/NM^+^).

#### Evaluation of pathological QC-positive structures

In addition to apparently intact neuronal cell bodies, QC immunoreactive, potentially pathogenic intra- and extracellular structures were identified and counted in the SNc. These were (1) degenerating neurons larger than 10 µm in diameter with very strong QC-immunoreactivity, which displayed a clearly aberrant form and/or were fractionated; (2) axonal varicosities; (3) axonal bulbs and Lewy neurites; (4) smaller (< 5 µm); and (5) larger (> 5 µm) Lewy body-like structures. QC-immunoreactive morphological features were termed axonal or dendritic *varicosities* when at least three punctate labellings smaller than 3 µm in diameter appeared along a neuronal process being arranged in a typical “beads on a string” manner, whereas slightly larger structures within axonal shafts (3–5 µm in diameter) were referred to as *axonal bulbs*.

#### Statistical analysis

The total number of identified neurons, as well as numbers of QC neurons and pathological structures in each category were compared between groups. Statistical analyses of the acquired data were performed by an unpaired *t* test. Differences between groups were considered statistically significant for *p* values < 0.05.

## Results

The enzymatic characterization of Gln79-α-synuclein conversion to the pGlu variant and the mass spectrometric analyses shown in Fig. 1c, d were carried out using a 12 amino acid peptide starting with Gln79 and termed α-synuclein79–90 and pGlu-α-synuclein79–90, respectively. The further analyses of aggregation properties and toxicity profiles were performed with recombinant pGlu79–140-α-synuclein, termed pGlu79-α-synuclein.

### QC-catalyzed generation of pGlu79-α-synuclein

To address the question whether Gln79-α-synuclein represents a QC substrate, the N-terminal part (79–90) of this α-synuclein fragment was synthesised and spontaneous as well as QC-catalyzed pGlu modification was followed by enzyme kinetic analysis (Fig. 1c). The subsequent calculation of kinetic parameters revealed a *V*_max_ of 0.5999 mM/h, *K*_m_ of 0.1333 mM and *K*_cat_ of 14.9/s. This is supportive for Gln79-α-synuclein being a QC substrate and compares well with other enzyme-catalyzed reactions, such as the conversion of the known physiological QC substrates gastrin, gonadotropin-releasing hormone and neurotensin [91].

Mass spectrometric analysis revealed that incubation of the α-synuclein79–90 fragment alone for up to 60 min did not lead to spontaneous pGlu modification of the N-terminus (Fig. 1d). By contrast, addition of QC to the incubation solution resulted in rapid pGlu-α-synuclein79–90 formation, which was already detectable after 10 min of incubation (Fig. 1d). By 1 h of incubation, the α-synuclein79–90 fragment was completely converted into pGlu-α-synuclein79–90. Addition of the QC inhibitor PBD150 prevented the pGlu modification (Fig. 1d).

### Aggregation characteristics and toxicity of pGlu79-α-synuclein

**Fig. 2:**
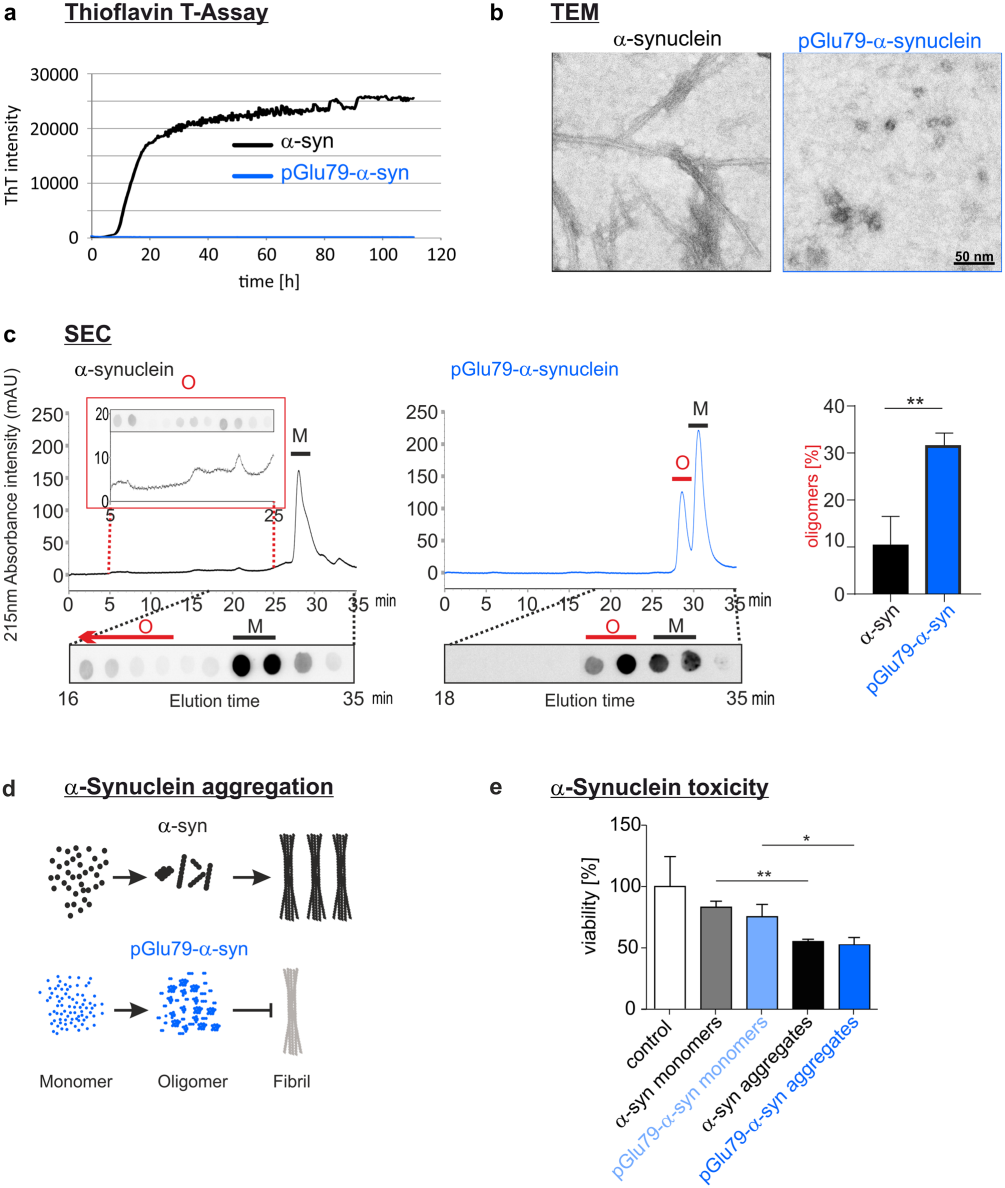
Aggregation characteristics of pGlu79-α-synuclein. **a** ThT assay to follow the characteristics of fibril formation from recombinant full-length α-synuclein and from pGlu79-α-synuclein. Note the typical, sigmoid-shaped curve of fibril formation from full-length α-synuclein (black trace) during the 110 h agitation period. In contrast, no fibril formation was observed for pGlu79-α-synuclein (blue trace). **b** Electron microscopic analysis of aggregates formed from full-length α-synuclein and from pGlu79-α-synuclein. Note the absence of fibril formation from pGlu79-α-synuclein but the presence of oligomers. **c** SEC and dot blot analysis of oligomers from full length or pGlu79-α-synuclein after 72 h agitation for protein aggregation. Agitated full-length and pGlu79-α-synuclein were centrifuged and the supernatants were analyzed by SEC. Their unagitated monomeric counterparts were also analyzed for determining the elution times of the monomers (Suppl. Figure 2, online resource). Peak elution times of full-length and pGlu79-α-synuclein monomers are 28.23 min and 30.74 min, respectively (see also Suppl. Figure 2, online resource). The representative chromatogram of agitated pGlu79-α-synuclein (right) demonstrates a remarkable increase in oligomers, characterized by a peak with an elution time of 29.93 min (indicated by O and red line), in addition to the peak for monomers at 31.86 min (M and black line). By contrast, the representative chromatogram of agitated full-length α-synuclein (left) is characterized by the presence of small peaks for oligomers with elution times between 5 and 25 min, however, to a much lesser extent (highlighted in the red inset), when compared to the peak for monomers at 29.40 min (black line). Dot blot analysis of SEC fractions confirmed the specificity of the peaks. For immunodetection, SEC fractions were either analyzed by the Syn1 antibody or the anti-pGlu79-α-synuclein antibody for detection of full-length α-synuclein and of pGlu79-α-synuclein, respectively. Quantification of three independent aggregation and SEC analyses shows significantly higher oligomer levels in agitated pGlu79-α-synuclein than in full-length α-synuclein. Statistical significance at **p < 0.01 defined by *t* test. **d** ThT assay and SEC, for analyzing fibril and oligomer formation, respectively, reveals that pGlu79-α-synuclein is more prone to form oligomers, however, is unable to form ThT-positive amyloid fibrils. **e** Analysis of cellular toxicity of monomers and aggregates of full-length α-synuclein (5 µM) and pGlu79-α-synuclein (5 µM). Cell viability was assessed by WST-1 assay in differentiated SH-SY5Y cells after 72 h of treatment with the peptides (mean ± SD, *n* = 3, *p < 0.05; **p < 0.01 defined by one-way ANOVA followed by Tukey post-hoc analysis)

The aggregation characteristics of recombinant pGlu79-α-synuclein were compared to those of full-length wild-type α-synuclein by continuous agitation of the α-synuclein variants and simultaneous monitoring of fibril formation by ThT assay. The full-length α-synuclein displayed a typical sigmoidal fibril formation behaviour (Fig. 2a). In contrast, no fibril formation from pGlu79-α-synuclein was detected by ThT assay (Fig. 2a). Transmission electron microscopy substantiated fibril formation from full-length α-synuclein and the presence of aggregates lacking fibrillary structures from pGlu79-α-synuclein (Fig. 2b). The labelled material analysed by electron microscopy represents most likely small oligomers, as shown for the maternal MMP-3-cleaved α-synuclein fragments [61, 102]. After 72 h of agitation, to confirm the formation of oligomers from pGlu79-α-synuclein, full-length and pGlu79-α-synuclein were centrifuged to remove the insoluble fibrils, and the supernatants were analysed by SEC (Fig. 2c). The chromatograms of agitated α-synuclein variants (Fig. 2c) were compared to the respective chromatograms of their monomers (Suppl. Figure 2, online resource). Indeed, SEC analysis of the soluble fraction of agitated pGlu79-α-synuclein revealed a remarkably increased formation of oligomers. The levels of oligomers formed from agitated pGlu79-α-synuclein were three times as high as those from full-length α-synuclein (Fig. 2c). Together, data from ThT assays, electron microscopy and SEC demonstrated that pGlu79-α-synuclein is prone to form oligomers, while full-length α-synuclein preferred forming ThT-positive amyloid fibrils (Fig. 2d).

To study the toxicity of full-length α-synuclein and pGlu79-α-synuclein, a WST-1 assay was performed using SH-SY5Y neuroblastoma cells. Monomers of both α-synuclein species and aggregates thereof produced by 72 h agitation were tested at concentrations of 5 µM and normalized to the vehicle control PBS. Compared to the monomeric peptides, a significant cytotoxic potential of aggregates of full-length α-synuclein and pGlu79-α-synuclein was observed (full-length α-synuclein monomers: 82% viability, full-length α-synuclein aggregates: 54% viability, pGlu79-α-synuclein monomers: 73% viability, pGlu79-α-synuclein aggregates: 50% viability, Fig. 2e).

### QC expression by SN dopaminergic neurons

**Fig. 3:**
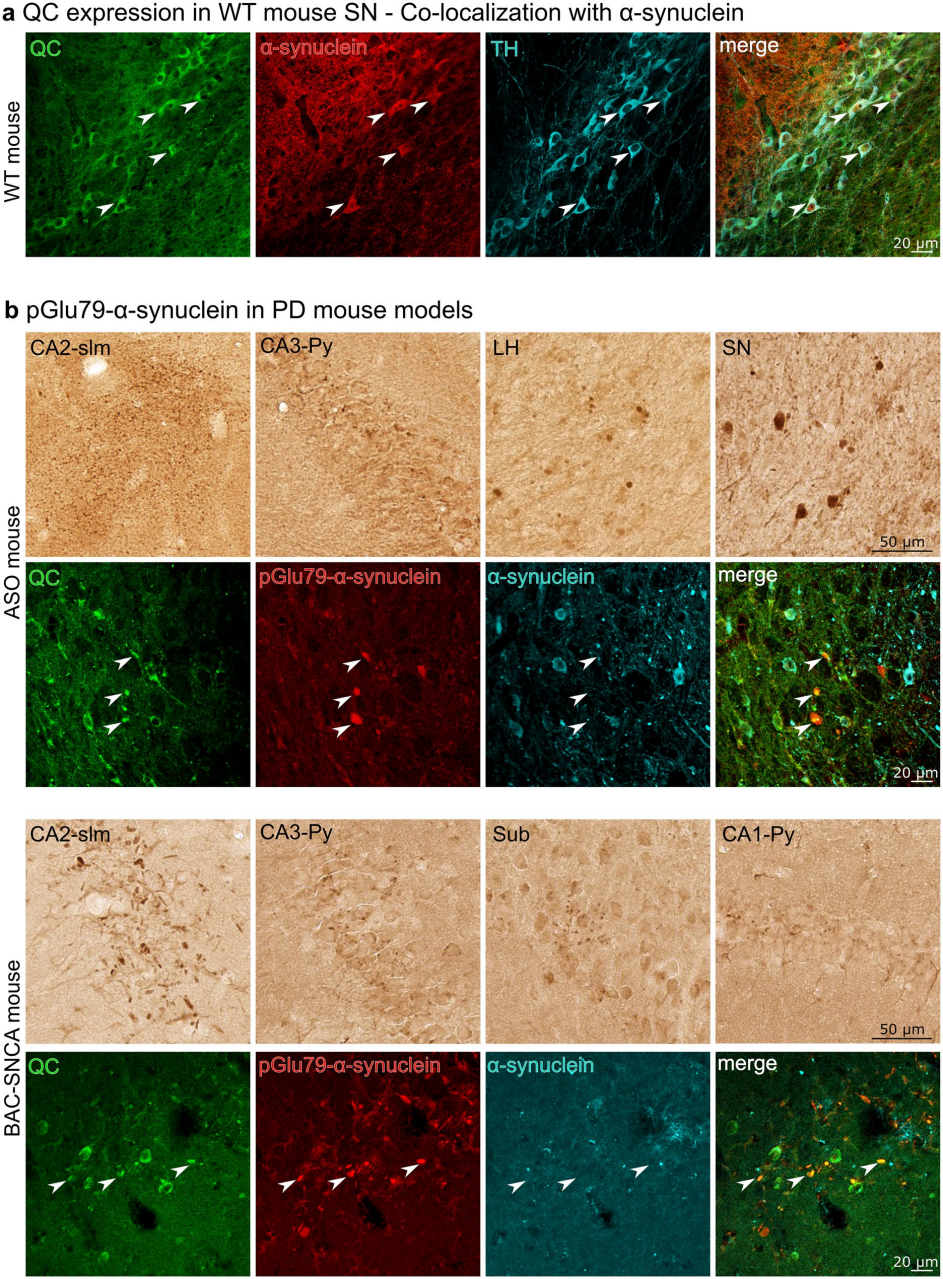
QC and pGlu79-α-synuclein in wild type and transgenic mouse brain. **a** QC expression by mouse TH-positive SN neurons and co-localization with α-synuclein. Triple immunofluorescent labellings demonstrate the expression of QC (green) by dopaminergic SN neurons (blue) and co-expression of maternal full-length α-synuclein (red) in mouse brain. **b** In brain sections of ASO mice (top), immunoreactivity for pGlu79-α-synuclein is displayed in fine, disperse aggregates in *stratum lacunosum* (slm) and pyramidal cell layer (Py) of the CA2 and CA3 hippocampal subregions, respectively, while larger aggregates and Lewy body-like structures are depicted in lateral hypothalamus (LH) and SN. Aggregates of pGlu79-α-synuclein in BAC-SNCA mice (bottom) are shown in hippocampus, either rod-shaped in CA2-slm or finely scattered in CA1-, CA3-Py and subiculum (Sub). Triple immunofluorescent labelling revealed a frequent co-localization of these aggregates with QC, as exemplarily shown for LH in ASO and for CA2 in BAC-SNCA mouse brain

The co-expression of QC and full-length α-synuclein by dopaminergic SN neurons is a prerequisite for pGlu79-α-synuclein formation in this brain region. Therefore, we first analyzed the co-expression of these proteins together with TH, the marker enzyme of dopaminergic neurons, in the SN of wild-type mouse brain sections by triple immunofluorescent labellings. As shown in Fig. 3a, QC is abundantly expressed by TH-positive neurons that also display α-synuclein immunoreactivity.

### pGlu79-α-synuclein aggregates in transgenic mouse models

Next, we wanted to test whether pGlu79-α-synuclein aggregates contribute to histopathology in brains of transgenic ASO and BAC-SNCA mice overexpressing human wild-type α-synuclein. Single pGlu79-α-synuclein DAB labellings revealed the presence of such aggregates in hippocampal and subcortical structures (Fig. 3b). In hippocampus, pGlu79-α-synuclein deposits were detected in all subregions. However, they were particularly prominent in CA2 *stratum lacunosum moleculare,* where densely dispersed labellings emerged in ASO mouse brain and where rod-shaped, neuritic structures appeared in BAC-SNCA mice. In addition, Lewy body-like aggregates were detected in lateral hypothalamus and SN in ASO mouse brain. Brain regions affected by these deposits differed between experimental animal models, which might be due to different transgene expression patterns. In both transgenic animal models, there was a frequent co-localization of QC and pGlu79-α-synuclein in these aggregates as exemplarily shown for lateral hypothalamus in ASO and for CA2 in BAC-SNCA mouse brain (Fig. 3b).

### pGlu79-α-synuclein aggregates in human substantia nigra

The main goal of our study was to reveal the presence and potential aggregation of pGlu79-α-synuclein in human clinical conditions of PD and DLB. Therefore, well-characterized high quality human brain tissue of short *post mortem* delay (1.5–4.2 h) was analyzed by immunohistochemistry. The dopaminergic SN neurons in human brain tissue can be easily identified by the intracellular presence of brown NM. However, this excludes the possibility of simultaneous immunohistochemical detection of intracellular antigens by brown DAB labelling in these neurons. We, therefore, visualized pGlu79-α-synuclein using DAB-Ni as histochemical substrate, resulting in black labelling.

**Fig. 4:**
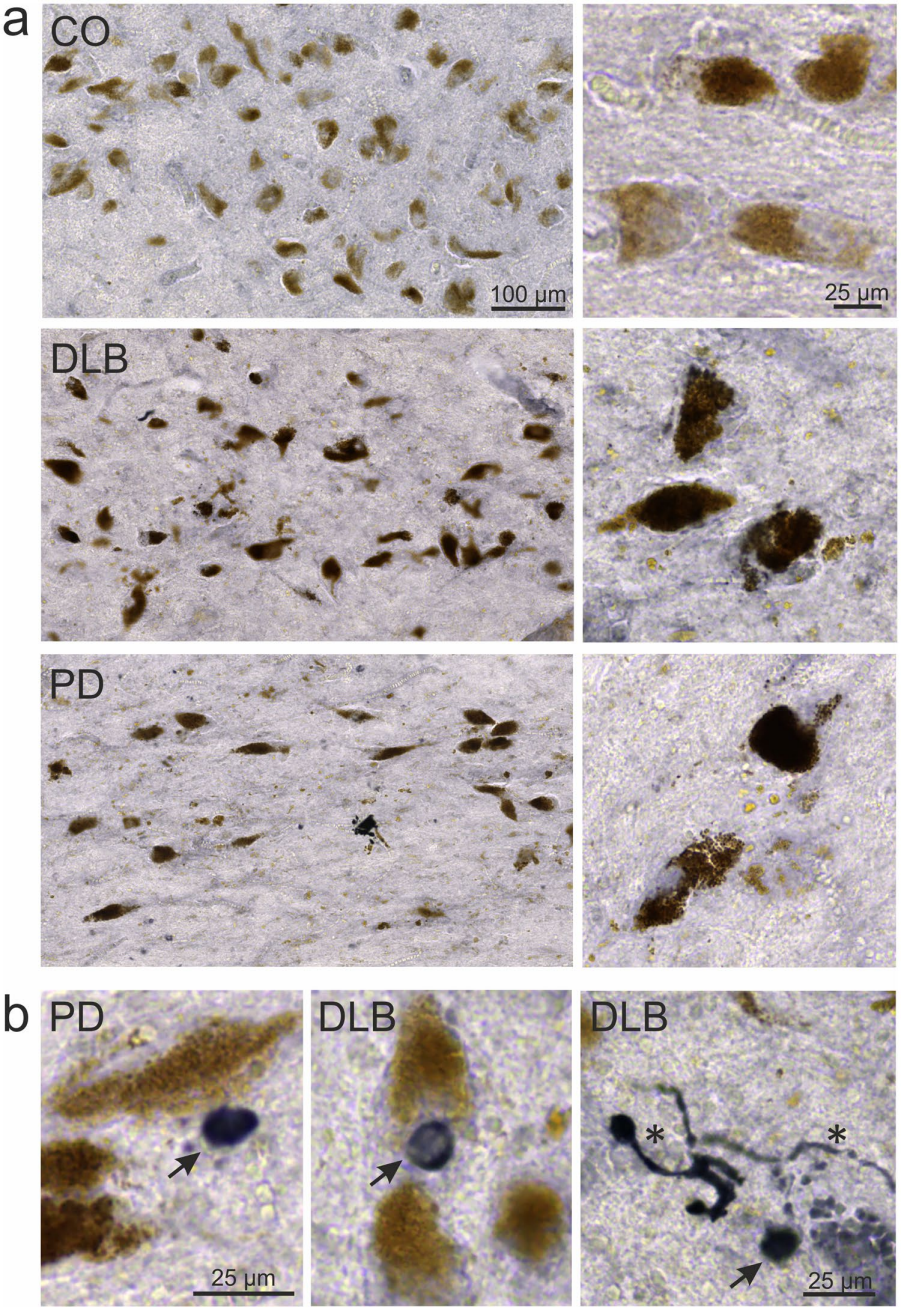
pGlu79-α-synuclein in human SN. **a** Typical examples of pGlu79-α-synuclein immunoreactivity in human SN of control subjects as well as PD and DLB patients. In control subjects, neuromelanin-containing SN neurons only sparsely contain pGlu79-α-synuclein (black DAB-Ni labelling). In PD and in DLB, the density of neuromelanin-containing neurons is markedly reduced, consistent with the degeneration of dopaminergic SN neurons in these clinical conditions. In addition, a high proportion of neuromelanin-positive neurons contains pGlu79-α-synuclein in PD and DLB cases. **b** pGlu79-α-synuclein immunoreactivity is also present in pathological structures, such as Lewy bodies (arrows) and Lewy neurites (asterisk) in PD and DLB cases

**Fig. 5:**
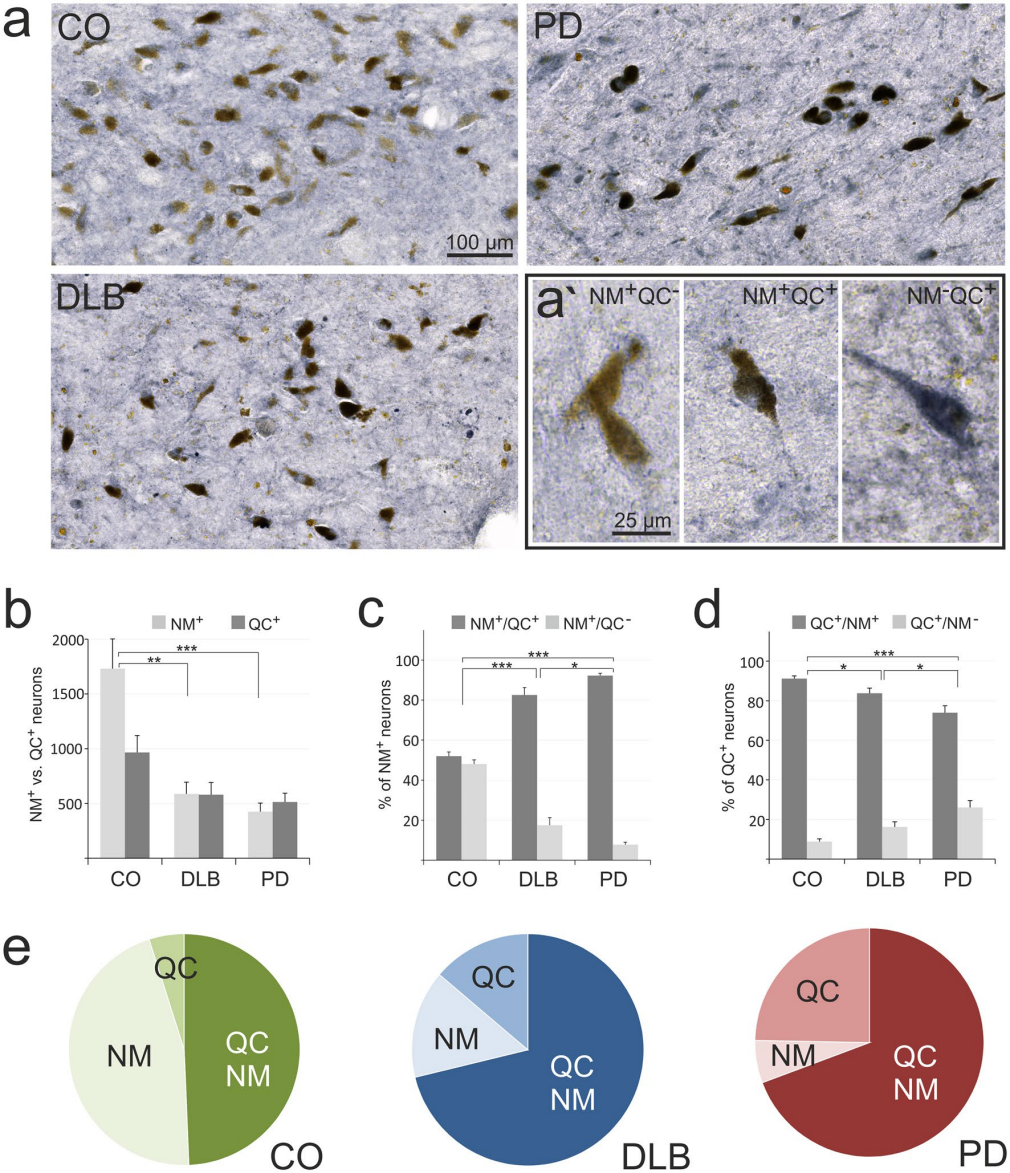
QC in human SN: Relation to neuromelanin. **a** Typical examples of immunohistochemical QC labellings (black) in SN of control, PD and DLB cases. Note the reduced numbers of neuromelanin-containing neurons (brown) in PD and DLB as compared to control and the differential association of QC (black) with brown, neuromelanin-containing neurons in the high magnification images (**a’**). **b** Quantification of all neuromelanin-containing (NM^+^) neurons (light bars) and NM^+^ neurons expressing QC (NM^+^/QC^+^; dark bars) illustrating the substantial loss of NM neurons in DLB and PD and the high proportion of NM^+^ neurons expressing QC in DLB and PD clinical conditions. **c** Proportions of NM^+^ neurons expressing QC (NM^+^/QC^+^) versus not expressing QC (NM^+^/QC^−^). Note the higher proportion of NM^+^ neurons expressing QC in DLB and PD clinical conditions. **d** Proportions of QC-expressing neurons associated with neuromelanin (QC^+^/NM^+^) versus not associated with neuromelanin (QC^+^/NM^−^). **e** Pie charts illustrating the drastically low proportion of NM^+^ only neurons in PD (6.1%) and in DLB (15.1%) compared to controls (45.7%), whereas the proportions of QC^+^ only and of NM^+^/QC^+^ neurons are increased in these groups. Mean ± SEM, n = 10, Statistical significance at *p < 0.05; **p < 0.005; ***p < 0.001 defined by *t* test

In human control subjects, only a small proportion of the numerous NM-positive neurons contained pGlu79-α-synuclein (Fig. 4). In PD and DLB cases, the number of NM-positive SN neurons was drastically reduced, consistent with the known degeneration of this cell group in the course of both clinical conditions (for quantification see Fig. 5). The remaining NM-containing neurons frequently displayed pGlu79-α-synuclein immunoreactivity and morphological signs of degeneration, such as shrinkage and irregular shape. In addition, PD-typical features, such as Lewy bodies and Lewy neuritis, were pGlu79-α-synuclein immunoreactive (Fig. 4). We conclude that a fraction of deposited α-synuclein in Lewy bodies and Lewy neurites consists of or contains pGlu79-α-synuclein.

### QC expression by human SN neurons: relation to neuromelanin

In analogy to the pGlu79-α-synuclein labelling, the expression of the enzyme QC catalyzing the pGlu modification was evaluated in human SN of control, PD and DLB cases (Fig. 5). Typically, in all conditions analyzed there were NM-containing neurons without QC immunoreactivity (NM^+^/QC^−^), NM-positive neurons expressing QC (NM^+^/QC^+^) and NM-negative neurons solely immunoreactive for QC (NM^−^/QC^+^) (Fig. 5a; highlighted in Fig. 5a’). As described above, the total number of NM-positive neurons (NM^+^) was much lower in DLB (587 ± 107 per brain section) and in PD (425 ± 77 per brain section) as compared to controls (1731 ± 270 per brain section) (Fig. 5b). In contrast, the numbers of QC^+^ neurons were only reduced from 964 ± 155 in controls to 580 ± 110 in DLB and to 512 ± 80 in PD (Fig. 5b).

When calculating the proportions of NM^+^/QC^+^ and of NM^+^/QC^−^ neurons in the various groups, controls displayed a 52% NM^+^/QC^+^ to 48% NM^+^/QC^−^ ratio, that was significantly different from DLB (82.5% to 17.5%) and from PD (92% to 8%) (Fig. 5c). Thus, in both clinical conditions a greater proportion of NM^+^ neurons displayed QC immunoreactivity.

From the perspective of all QC immunoreactive neurons, 91% were associated with NM in control SN, whereas this proportion was reduced to 84% in DLB and to 74% in PD (Fig. 5d). This was accompanied by a concomitant increase in the proportion of QC^+^/NM^−^ neurons from 9% in controls to 16% in DLB and 26% in PD (Fig. 5d). Out of these, a subgroup of morphologically degenerating and often shrunken or fractionated neurons stood out, which displayed excessive QC immunoreactivity (see below).

The summary pie charts (Fig. 5e) illustrate the drastic reduction of the proportion of NM^+^/QC^−^ neurons in DLB (15%) and in PD (6%) compared to controls (46%), whereas the proportions of QC^+^/NM^+^ and of QC^+^/NM^−^ neurons are increased.

### QC in human SN: presence in neuropathological structures

**Fig. 6:**
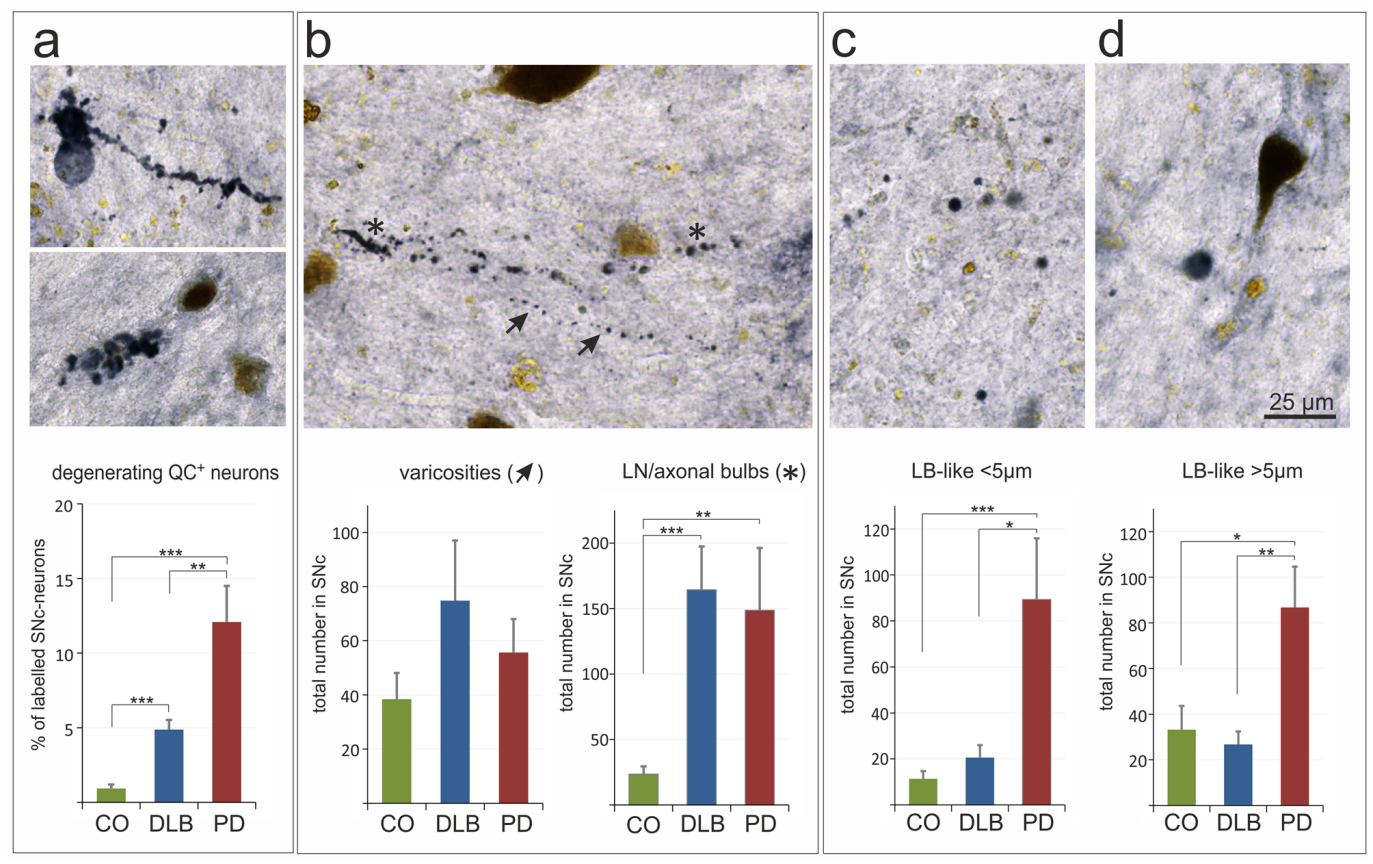
QC in human SN: presence in neuropathological structures. QC (labelled in black) was found to be associated with degenerating neurons (**a**), with varicosities (arrows) and Lewy neurites/axonal bulbs (asterisks) (**b**), as well as with small (**c**) and large (**d**) Lewy body-like structures. The quantification of these structures revealed a high association of QC with Lewy body-like aggregates in PD, but not in DLB (**c**, **d**) no significant differences in the number of QC-positive varicosities between groups, but a similarly strong increase in the number of QC immunoreactive Lewy neurites and axonal bulbs in DLB and PD compared to controls (CO) (**b**), and a more abundant association of QC with degenerating neurons in PD than in DLB (**a**). Brown color arises from neuromelanin. Mean ± SEM, *n* = 10, Statistical significance at *p < 0.05; **p < 0.01; ***p < 0.001 defined by *t*-test

QC was not only found to be associated with NM-positive neurons in the SN, but was additionally detected in typical neuropathological structures of synucleinopathies. These structures include degenerating neurons (Fig. 6a), axonal or dendritic varicosities and Lewy neurites/axonal bulbs (Fig. 6b), as well as Lewy body-like aggregates, smaller and larger than 5 µm in diameter (Fig. 6c, d). The corresponding quantifications demonstrate a much higher abundance of QC-immunoreactive degenerating neurons in DLB (4.9%) and in PD (12.1%) than in control conditions (0.9%) (Fig. 6a). While the numbers of QC-immunoreactive varicosities did not differ between clinical groups, counts of QC-immunoreactive Lewy neurites/axonal bulbs in DLB (164 ± 33) and PD (149 ± 48) were significantly higher compared to controls (23 ± 6) (Fig. 6b). Interestingly, both, the numbers of QC-immunoreactive Lewy body-like aggregates of smaller and of larger size, were significantly higher in SN of PD, but not of DLB, subjects compared to controls (Fig. 6c, d). This is consistent with the more cortical pathology of DLB as compared to the SN pathology in PD [51].

However, it should be noted that these QC-immunoreactive structures were also present in control subjects; although to a much lower extent. These aggregates, therefore, may represent early pathological events in clinically silent subjects.

## Discussion

### Formation of pGlu79-α-synuclein by QC

Post-translational modifications of α-synuclein have been extensively studied with respect to the regulation of its physiological functions and their contribution to pathological processes in clinical conditions, such as PD and DLB [17, 37]. The most prominent disease-related modification is α-synuclein phosphorylation at serine129 [4, 32, 86], which could become an early peripheral diagnostic marker [1, 26, 30, 67, 104].

In the present study, we focused on proteolytically cleaved fragments of α-synuclein. MMP-3 cleavage of α-synuclein generates a number of defined fragments with distinct biophysical and cell biological properties [80, 102], for review see [13, 98]. One of the identified MMP-3-generated α-synuclein fragments, Gln79-α-synuclein, possesses a glutamine residue at its N-terminus and could, therefore, serve as QC substrate. In cell free assays, we demonstrate QC-catalyzed conversion of this fragment into the pGlu-modified form that follows kinetic characteristics of enzyme-catalyzed reactions (see Fig. 1). When we compared the aggregation behavior of pGlu79-α-synuclein to that of maternal, full-length α-synuclein applying ThT assays, we noticed a lack of fibril formation from pGlu79-α-synuclein. This is consistent with the loss of a significant part of the NAC domain and observations by others, analyzing the aggregation behavior of different α-synuclein fragments [33, 61, 102]. Cryo-EM structures of full-length α-synuclein reveal that various residues (46–95) are associated with fibril formation and stabilization by the generation of hydrophobic cores of a single protofilament and steric zippers of two protofilaments [39, 62]. Since most of these residues critical for fibril stabilization are absent in pGlu79-α-synuclein, fibrillation is most likely disabled and aggregation is limited to the oligomeric state. In addition, it is speculated that fibril elongation by hydrophobic interaction might be related to residues V74–V82, because β-synuclein lacking these residues is incapable of forming fibrils [36]. However, the lack of fibril formation does not exclude the generation of oligomeric α-synuclein assemblies after MMP-3 cleavage as established in two independent studies employing different analytical methods [61, 102]. Indeed, uncleaved α-synuclein oligomers have been shown to be a neurotoxic species in vivo [84, 107] and, intriguingly, α-synuclein oligomers generated following processing by MMP-3 have also been shown to exert a more toxic effect on cultured cells than oligomers composed of full-length α-synuclein [61, 102]. Here, using ThT assay and size exclusion chromatography, we demonstrate distinct aggregation behaviors of full-length and pGlu79-α-synuclein. Under continuous agitation, a classic in vitro condition for fibrillization, a large fraction of full-length α-synuclein is converted into amyloid fibrils, whereas pGlu79-α-synuclein is prone to form soluble oligomers. The lack of formation of ThT positive amyloid fibrils of the latter suggests an off-pathway in the aggregation of this cleaved and modified α-synuclein species. These observations are also reflected in the cytotoxicity assay. Although pGlu79-α-synuclein does not tend to form fibrils, its toxicity on neuroblastoma cells is comparable to aggregates of full-length α-synuclein. This is in line with evidence that MMP-3 knock-out mice display attenuated neuronal death in the MPTP mouse PD model in vivo [56]. Moreover, prion-like seeding of misfolded α-synuclein is propagated from oligomer-like species, but not from insoluble aggregates [11, 27, 87] and α-synuclein oligomers were reported to stabilize pre-existing defects in supported bilayers and propagate membrane damage [19]. In living cells, the stabilization of α-synuclein oligomers resulted in increased cytotoxicity, which could be rescued by Hsp70 via suppression of oligomer formation [77]. Thus, oligomer formation from α-synuclein fragments lacking parts of the NAC domain is likely to lead to pathogenic protein assemblies.

### Detection of pGlu79-α-synuclein in transgenic animal models

If QC-catalyzed modification of α-synuclein does occur in vivo, the maternal α-synuclein and QC should be co-localized. QC is not ubiquitously distributed throughout the brain but is rather restricted to defined regions, such as hypothalamus and pituitary, where physiological substrates reside [14, 43]. In addition, QC is abundant in brain nuclei, such as nucleus basalis Meynert, locus coeruleus and Edinger–Westphal nucleus, which are affected by amyloid pathology in AD [45, 73]. Here, we demonstrate co-expression of α-synuclein and QC by TH-positive dopaminergic SN neurons in brain of wild-type mice.

In addition, two transgenic mouse lines with overexpression of human α-synuclein—ASO and BAC-SNCA—were analyzed. Aged BAC-SNCA mice have a 2.7-fold amount of endogenous α-synuclein and show highest transgene expression levels in cortex, striatum and hippocampus [108]. They display a high abundance of monomeric α-synuclein but also higher order SDS-resistant α-synuclein [71]. In ASO mice, Thy-1-driven transgene expression results in 1.5–3.4-fold overexpression of α-synuclein in many brain regions including olfactory bulb, SN, thalamus, cortex, hippocampus and striatum [20]. Already in 5-month-old transgenic mice, proteinase K-resistant α-synuclein aggregates are present in SN and oligomeric assemblies were detected by Western blot analysis [20]. The transgene is inserted in the X chromosome, which leads to diminished motor deficits in females, most likely due to random inactivation of the X chromosome carrying the mutation [20]. Therefore, only male ASO mice were used in the present study.

When detecting pGlu79-α-synuclein in both α-synuclein overexpressing mouse models, we found increased immunoreactivity in subcellular sites of physiological α-synuclein presence, such as synapses, confirming the specificity of the antibody. Focusing on histopathological features, we identified a co-localization of QC and pGlu79-α-synuclein in Lewy body-like structures predominantly in ASO mice and in disperse aggregates in brain tissue of both mouse models. These aggregates were found in brain structures that are known for the formation of α-synuclein deposits in these animal models but also in different hippocampal subregions, such as subiculum, CA1, CA3 and pronounced in the *stratum lacunosum* sublayer of CA2. Distinct hippocampal CA2 synuclein pathology has been associated with cholinergic degeneration in PD with cognitive decline [64]. Thus, there is co-localization of QC with α-synuclein in SN of mouse brain and a spatial association of both proteins in protein aggregates of transgenic animal models of synucleinopathies with relevance to the clinical condition in human patients.

### Localization of QC and pGlu79-α-synuclein in human brains affected by synucleinopathies

Animal models for protein aggregation disorders do not always reflect the exact scenario present in human disease. Therefore, it is mandatory to validate observations derived from experimental animals in human brain tissue. Here, using well-characterized human brain tissue sections from control subjects as well as PD and DLB patients, we demonstrate robust deposition of pGlu79-α-synuclein in SN in a disease-specific manner. The detection of pGlu79-α-synuclein in Lewy bodies and Lewy neurites is reminiscent of the classical α-synuclein aggregates representing typical pathological hallmarks in Lewy body diseases, such as PD and DLB [16, 88]. The synucleinopathy typically affects NM-containing dopaminergic neurons of the SN complex [48, 69, 96]. The precise biochemical composition of Lewy bodies has not yet been decoded. However, in addition to the main component full-length α-synuclein, the presence of truncated fragments [110] and of pSer129-α-synuclein [4] has been reported. Here, we show for the first time pGlu79-α-synuclein immunoreactivity in neuromelanin positive SN neurons and accumulation in Lewy bodies and Lewy neurites. The post-translational α-synuclein modification present in this fragment is catalyzed by QC, which we demonstrated to be associated with neuropathological structures in SN of PD and DLB subjects in addition to its expression in neuronal somata. It was not in the scope of this study to define the exact nature of the varieties of QC- and pGlu79-α-synuclein-immunoreactive axonal and parenchymal neuronal structures with pathological appearance. Descriptions of the morphology and nature of small pathogenic alterations and even the subcellular localization of varicosities, axonal or dendritic, in human PD and DLB brain tissue differ considerably in the literature [78, 103], for review see [38].

Interestingly, the proportion of NM-containing SN neurons that express QC is increased in PD and DLB (see Fig. 5e), indicating a contribution of QC to both disorders. This finding can be interpreted in two different ways. On the one hand one could reason that neuronal degeneration mainly affects the subset of NM^+^ neurons which is devoid of QC, assuming that its presence in surviving neurons is neuroprotective. On the other hand QC expression may be newly induced in a neuronal subfraction of SNc in the course of the disease, rendering the respective neurons more vulnerable to the pathological process.

In PD brains, compared to the DLB or the control group, we observed a statistically significant increase in the actual number of neurons in the SNc that solely express QC without containing neuromelanin. This can only be explained by de novo synthesis of QC in a substantial fraction of this neuronal category (QC^+^/NM^−^). Furthermore, the percentage of degenerating neurons with excessively high QC immunoreactivity is augmented five and twelve times, respectively, in DLB and PD cases in comparison to control subjects (Fig. 6a). Taken together, it appears most likely that QC expression by SNc neurons is a factor which contributes to neuropathology rather than to neuroprotection. However, while the incidence of QC-positive structures in Lewy neurites and varicosities is comparable in PD and DLB, the association of QC with Lewy body-like structures in SN was more pronounced in PD than in DLB (Fig. 6), which is consistent with a more cortical pathology in DLB [51]. It would be interesting to reveal whether the same type of association between QC and pGlu79-α-synuclein and neuropathological structures is also present in other types of synucleinopathies, such as multiple system atrophy or Alzheimer’s disease with amygdala-restricted Lewy bodies that were not analyzed here.

We propose that QC-catalyzed pGlu-modification of pathogenic proteins might be a more general mechanism in human clinical conditions than previously thought. In AD, it is well established that the pGlu modification of Aβ peptides initially described by the Saido and Roher groups [58, 85] compromises Aβ degradation and increases its toxicity [76] as well as aggregation propensity in human cortex, hippocampus and subcortical brain nuclei affected by amyloid pathology [42, 72, 73]. There are currently two independent therapeutic strategies being tested in preclinical studies and in clinical settings: (1) inhibition of QC to prevent pGlu-Aβ formation and (2) targeting existing amyloid assemblies by pGlu-Aβ-specific antibodies.

Pharmacological inhibition of QC activity and genetic ablation of QC in transgenic animal models reduced pGlu-Aβ generation and total Aβ load and ameliorated learning and memory deficits [3, 44, 50, 93]. In addition, clinical trials indicated safety, tolerability and efficacy of the QC inhibitor PQ912 in human subjects [65, 89]. For information on ongoing clinical trials, see https://www.alzforum.org/therapeutics/varoglutamstat.

Targeting pGlu-Aβ using a well-characterized monoclonal antibody [79] reduced amyloid plaques and improved cognition in APP transgenic mice [22, 31, 49]. Using this specific passive immunization approach, an attenuated cognitive decline was reported in people with early AD in the Phase 2 TRAILBLAZER-ALZ study (https://www.alzforum.org/news/research-news/phase-2-donanemab-curbs-cognitive-decline-early-alzheimers).

Thus, there are well-characterized tools with defined cell biological, pharmacological and safety properties already available that can be tested in cellular and animal models of synucleinopathies in a straightforward way, to interfere with pGlu79-α-synuclein generation and to test its contribution to motor, cognitive and histological disturbances.

## Conclusions

We demonstrate the QC-catalyzed formation of pGlu79-α-synuclein, its oligomerization and neurotoxic profiles and its accumulation in brain in two transgenic mouse models of synucleinopathy. In human brain, the presence of QC and pGlu79-α-synuclein is largely increased in neurons of the SN and associated with pathological structures in PD and DLB subjects. Given the resistance of pGlu-modified proteins against proteolytical degradation in general and the high oligomer formation velocity of pGlu79-α-synuclein in particular, these molecules might be interesting novel targets for pharmacologically interfering with human synucleinopathies.

## Supplementary Information

Below is the link to the electronic supplementary material.

Supplementary file1 (PDF 469 kb)

## Data Availability

The data sets used and analyzed during the current study are available from the corresponding author on reasonable request.

## Abbreviations

AD: Alzheimer’s disease
BSA: Bovine serum albumin
DAB: 3,3′-Diaminobenzidine
DLB: Dementia with Lewy bodies
DMF: Dimethyl formamide
EDT: 1,2-Ethanedithiol
ESI: Electrospray ionization
MMP: Matrix metalloproteinase
NAC: Non-Abeta component
NM: Neuromelanin
PBS: Phosphate-buffered saline
PD: Parkinson’s disease
QC: Glutaminyl cyclase
SN: Substantia nigra
SNc: Substantia nigra pars compacta
TBS: Tris-buffered saline
TFA: Trifluoroacetic acid
TH: Tyrosine hydroxylase
ThT: Thioflavin T
TIS: Triisopropylsilane
