# Supplementary material for: A glutaminyl cyclase-catalyzed α-synuclein modification identified in human synucleinopathies
Source: Acta Neuropathol. 2021 Jul 26;142(3):399–421. doi: 10.1007/s00401-021-02349-5 (PMC8357657; doi:10.1007/s00401-021-02349-5)
Supplement: Supplementary file 1 — Supplementary file1 (PDF 469 kb) [file 401_2021_2349_MOESM1_ESM.pdf]

Supplementary Information to

A glutaminyl cyclase-catalyzed α-synuclein modification identified in human synucleinopathies

Maike Hartlage-Rübsamen[1], Alexandra Bluhm[1], Sandra Moceri[2], Lisa Machner[3], Janett Köppen[3], Mathias Schenk[3], Isabel Hilbrich[1], Max Holzer[1], Martin Weidenfeller[4], Franziska Richter[5], Roland Coras[6], Geidy E. Serrano[7], Thomas G. Beach[7], Stephan Schilling[3], Stephan von Hörsten[2], Wei Xiang[4], Anja Schulze[3], Steffen Roßner[1§]

1 Paul Flechsig Institute for Brain Research, University of Leipzig, 04103 Leipzig, Germany

2 Department for Experimental Therapy, Universitätsklinikum Erlangen, and Preclinical Experimental Animal Center, Friedrich-Alexander-Universität Erlangen-Nürnberg, 91054 Erlangen, Germany

3 Fraunhofer Institute for Cell Therapy and Immunology, Department of Molecular Drug Design and Target Validation, 06120 Halle (Saale), Germany

4 Department Molecular Neurology, Universitätsklinikum Erlangen, Friedrich-Alexander-Universität Erlangen-Nürnberg, 91054 Erlangen, Germany

5 Department of Pharmacology, Toxicology, and Pharmacy, University of Veterinary Medicine Hannover, 30559 Hannover, Germany

6 Institute for Neuropathology, Universitätsklinikum Erlangen, Friedrich-Alexander-Universität Erlangen-Nürnberg, 91054 Erlangen, Germany

7 Civin Laboratory for Neuropathology Brain and Body Donation Program Banner Sun Health Research Institute, 10515 W Santa Fe Drive, Sun City, AZ 85351, USA

Submitted to: Acta Neuropathologica

Demonstration of pGlu79-α-synuclein antibody specificity

The specificity of the affinity-purified rabbit polyclonal pGlu79-α-synuclein antiserum is of highest importance for the validation of data on the presence and cellular localization of pGlu79-α-synuclein in brain of experimental animals and human subjects and for the conclusions drawn on the significance of this α-synuclein modification in human synucleinopathies. Therefore, rigorous tests were performed using recombinant synuclein proteins for dot blot analysis and brains of α-synuclein overexpressing and knock-out (KO) mice for immunohistochemistry.

Affinity-purified recombinant human full-length α-synuclein, β-synuclein, γ-synuclein and the target pGlu79-α-synuclein fragment were spotted at descending amounts ranging from 500 ng to 31.25 ng onto nitrocellulose membranes. After chemiluminescent detection using the pE79-synuclein antiserum (1:1,000) and donkey anti-rabbit peroxidase conjugates (Sigma; 1:5,000), membranes were stripped and re-probed with the α-synuclein-specific Syn1 antibody (BD Transduction; 1:2,000). The pGlu79-α-synuclein antiserum detected its target protein but not full-length α-synuclein nor β-synuclein or γ-synuclein proteins (**Suppl. Fig. 1a)**. The Syn1 antibody detected both, its target protein α-synuclein, but also – with lower specificity – the pGlu79-modified α-synuclein. It did not show immunoreactivity against β-synuclein and γ-synuclein (**Suppl. Fig. 1a**).

Immunohistochemical labellings on wild type (WT), α-synuclein overexpressing (ASO) and α-synuclein KO mouse brain sections were performed to reveal the specificity of the pGlu79-α-synuclein antiserum for this application. Immunohistochemistry was performed as described in the main manuscript text. The labelling generated by the pGlu79-α-synuclein antiserum was stronger in ASO mouse brain sections compared to WT mouse (**Suppl. Fig. 1b**). In α-synuclein KO mouse brain sections, only unspecific background staining was present, but the labelling of defined structures was absent. A similar immunohistochemical labelling between genotypes was observed for the Syn1 antibody (**Suppl. Fig. 1b**).

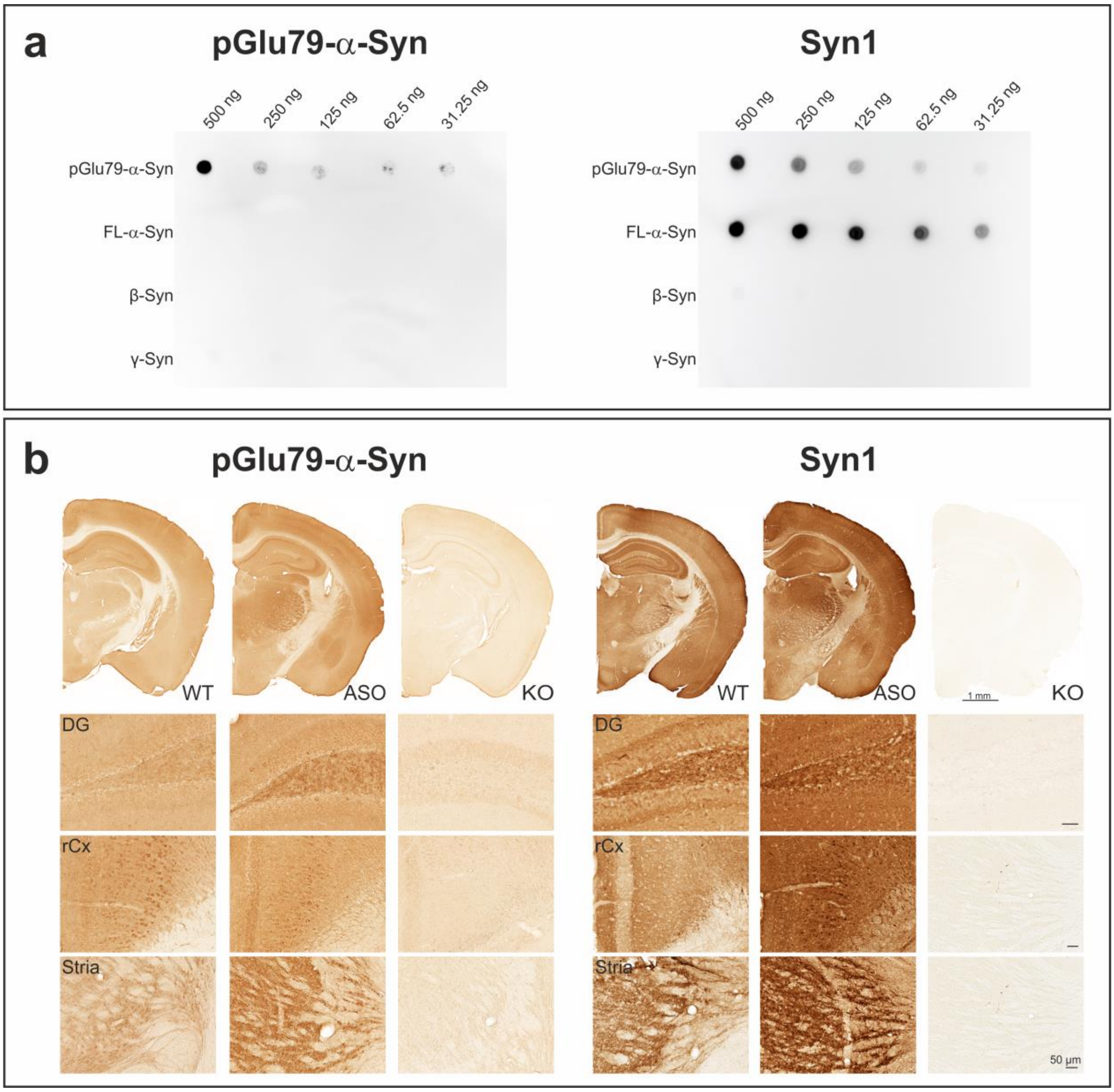

Suppl. Fig. 1: pGlu79-α-synuclein antiserum specificity

(a) Dot blot analysis to detect pGlu79-α-synuclein, full-length (FL) α-synuclein, β-synuclein and γ-synuclein spotted at different amounts as indicated onto nitrocellulose membranes. *Left:* Detection using the pGlu79-α-synuclein antiserum; *Right:* Detection using the monoclonal Syn1 antibody. Note the specificity of the pGlu79-α-synuclein antiserum for its target protein and the detection of both, pGlu79-α-synuclein and FL α-synuclein by Syn1.

(b) Immunohistochemistry on mouse brain sections from wild type (WT), α-synuclein overexpressing (ASO) and α-synuclein knock-out (KO) mice. Note the defined labelling of distinct structures in WT mouse brain sections, the stronger labelling in ASO brain sections and the absence of specific labelling KO brain sections for both antibodies in the low magnification images (top) and at higher magnification in dentate gyrus (DG), retrosplenial cortex (rCx) and striatum (Stria).

SEC analysis of full-length and pGlu-α-synuclein monomers. The elution time for the full-length α-synuclein was 28.23 minutes, compared to 30.74 minutes for the pGlu79-α-synuclein (**Suppl. Fig 2**).

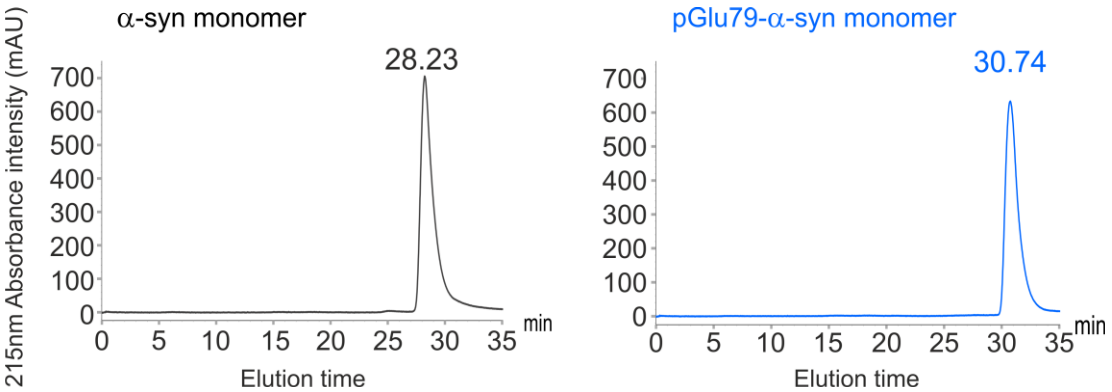

Suppl. Fig. 2: Example chromatograms of 50 µg full-length α-synuclein and pGlu79-α-synuclein monomers used for SEC. The full-length and pGlu79-α-synuclein monomers were eluted at 28.23 min and 30.74 min, respectively.
